# The gait disorder in primary orthostatic tremor

**DOI:** 10.1007/s00415-020-10177-y

**Published:** 2020-09-11

**Authors:** Ken Möhwald, Max Wuehr, Fabian Schenkel, Katharina Feil, Michael Strupp, Roman Schniepp

**Affiliations:** 1 German Center for Vertigo and Balance Disorders, University Hospital, LMU Munich, Munich, Germany; 2 Department of Neurology, University Hospital, LMU Munich, Marchioninistr. 15, 81377 Munich, Germany

**Keywords:** Orthostatic tremor, Gait disorder, Walking stability, Gait ataxia

## Abstract

**Objective:**

To uncover possible impairments of walking and dynamic postural stability in patients with primary orthostatic tremor (OT).

**Methods:**

Spatiotemporal gait characteristics were quantified in 18 patients with primary OT (mean age 70.5 ± 5.9 years, 10 females) and 18 age-matched healthy controls. One-third of patients reported disease-related fall events. Walking performance was assessed on a pressure-sensitive carpet under seven conditions: walking at preferred, slow, and maximal speed, with head reclination or eyes closed, and while performing a cognitive or motor dual-task paradigm.

**Results:**

Patients exhibited a significant gait impairment characterized by a broadened base of support (*p* = 0.018) with increased spatiotemporal gait variability (*p* = 0.010). Walking speed was moderately reduced (*p* = 0.026) with shortened stride length (*p* = 0.001) and increased periods of double support (*p* = 0.001). Gait dysfunction became more pronounced during slow walking (*p* < 0.001); this was not present during fast walking. Walking with eyes closed aggravated gait disability as did walking during cognitive dual task (*p* < 0.001).

**Conclusion:**

OT is associated with a specific gait disorder with a staggering wide-based walking pattern indicative of a sensory and/or a cerebellar ataxic gait. The aggravation of gait instability during visual withdrawal and the normalization of walking with faster speeds further suggest a proprioceptive or vestibulo-cerebellar deficit as the primary source of gait disturbance in OT. In addition, the gait decline during cognitive dual task may imply cognitive processing deficits. In the end, OT is presumably a complex network disorder resulting in a specific spino-cerebello-frontocortical gait disorder that goes beyond mere tremor networks.

## Introduction

Primary orthostatic tremor (OT) is a rare neurological disease, characterized by a high-frequency tremor with a typical 13–18 Hz pattern affecting the leg and trunk musculature [1]. The tremor, which is associated with the patient’s feeling of unsteadiness and dizziness, predominantly occurs during standing and vanishes in most patients while sitting or lying [2, 3]. Postural impairments in OT are, thus, primarily associated with static postural control. It is less clear whether and how OT affects dynamic postural control during walking. While most patients report a relief of symptoms when starting to ambulate, tremor activity has been recently shown to persist even during walking [4]. Ongoing high-frequency tremor activity might interfere with both central as well as peripheral feedback control of gait in OT patients. First, the pathological central oscillatory network activity that has been associated to OT encompasses supraspinal locomotor regions in the cerebellum, thalamus and motor cortex [5]. Second, high-frequency tremor bursts in the legs were shown to considerably impair afferent proprioceptive signaling in patients with OT [6]. It is, thus, conceivable, that OT is associated with a specific gait disorder as known in other types of tremor, such as essential tremor [7]. Our previous analyses depicted gait anomalies with broad-based walking and increased gait fluctuations resembling a cerebellar or sensory ataxic gait impairment [4]. However, gait performance was assessed in a highly controlled setting of treadmill walking considering only a limited set of gait conditions.

Therefore, the aim of the study was to comprehensively characterize the gait abnormalities associated to OT by means of a quantitative examination of gait performance in patients with OT compared to healthy controls utilizing a standardized multiple condition gait assessment.

## Methods

### Ethics approval and patients’ consent

The study procedures have been approved by the Ethics Committee of the University of Munich. All procedures were in accordance with the Declaration of Helsinki and all patients gave their written informed consent.

### Participants and clinical assessment

Eighteen patients with primary OT (mean age 70.5 ± 5.9 years, 10 females) with typical symptoms and high-frequency tremor as well as 18 age-matched healthy controls (mean age 71.2 ± 6.3 years, 10 females) participated in the study. The patient cohort was derived from a previous study from our medical center focusing on the course of disease and postural imbalance assessed by posturographic measurements [8]. The average disease duration of OT was 14.0 ± 7.0 years. Detailed patient characteristics are presented in Table 1. All patients underwent a standardized neurological, neuro-ophthalmological, and neuro-otological examination. In addition to the neurological evaluation, this included assessment of eyesight and eye movements (smooth pursuit, spontaneous, gaze-evoked and head-shaking nystagmus, impaired visual fixation, among others), a head impulse test and examination of the subjective visual vertical (SVV). A cerebellar ocular motor disorder was defined by the presence of one of the following findings: gaze-evoked nystagmus (left, right, both horizontally) or downbeat nystagmus, rebound nystagmus, impaired visual fixation suppression of the VOR [9]. Pallesthesia was assessed using a C64/128 Hz tuning fork. Reduced vibration sense was defined as pallesthesia ≤ 4/8. Romberg’s test was considered positive, when patients showed signs of imbalance with considerable staggering after eye closure or if they were unable to perform the task. All patients completed the Activities-specific Balance Confidence Scale (ABC, ranging from 0 to 100%, > 80 high level of physical function; 50–80 moderate level of physical function; < 50 low level of physical function) and the Falls Efficacy Scale—International (FES-I, total score range from 16, i.e., no concern about falling, to 64, i.e., severe concern about falling) and were screened for fall events within the last 6 months.

**Table 1:** Patient characteristics including neurological findings and medication

Patient	Gender	Age (years)	Tremor frequency (Hz)	Duration (years)	Tremor treatment	Cerebellar signs	Further neurological findings	ABC	FES-I	FGA	Falls
1	f	54	17.0	7	Baclofen 30 mg/day	3	1, 6, 7	33	39	18^a^	No
2	f	73	16.5	26	Primidone 125 mg/day	1	1, 5, 6	22	42	19	Yes
3	m	77	17.5	23		1, 2, 3	1, 5, 6, horizontal periodic alternating nystagmus	43	43	20	No
4	m	72	13.5	23		3	1, 2, 4, 5, 6	15	51	15^a^	No
5	f	62	16.0	15		2, 3	1, 5, 6, 7	49	40	17^a^	Yes
6^c^	m	72	14.5	16	Clonazepam 0.5 mg/day	1, 2, 3	1, 2, 5, 6	53	28	18	Yes
7	f	72	17.0	8	Gabapentin 1200 mg/day + Primidone 375 mg/day	1, 2, 3	1, 2, 4, 5, 6	7	57	^b^	Yes
8	f	63	14.0	15	Pregabalin 150 mg/day	1, 2	1, 4, 5, 7	59	21	25	No
9	f	69	15.0	19	Gabapentin 300 mg/day	1, 2, 3	1, 4, 5, 6, head tremor	37	37	16^a^	No
10	m	72	17.0	10		2	1, 4, 5	87	25	26	No
11^c^	f	78	14.0	18	Gabapentin 900 mg/day	1, 2, 3	1, 2, 5, 6	62	46	^b^	Yes
12	f	72	16.0	20		2	1, 5, 6, pronation forearm holding test	24	48	18	No
13	m	77	14.5	18	Gabapentin 2000 mg/day	1, 2	1, 2, 5, 6	75	28	12^a^	No
14	m	73	11.5	8		2, 3	1, 5, 6, muscle atrophy of lower limb (right > left)	65	28	19	No
15	m	71	13.0	1		1, 2, 3, 4	1, 2, 5, 6, 7	24	24	20	No
16	f	72	11.0	5	Propranolol 120 mg/day	1, 4	1, 6, pronation forearm holding test	58	30	17	Yes
17	m	73	14.0	11		1, 2, 3	1, 2 (right), 3, 4, 5, 6	28	49	9^a^	No
18	f	67	15.5	9	Clonazepam 0.3 mg/day	1, 2	1, 2, 4, 5, 6, drooping mouth on right side	81	25	^b^	No

Cerebellar signs: 1 = cerebellar ocular motor disorder, 2 = upper limb dysmetria, dysdiadochokinesia or intention tremor (uni- or bilateral), 3 = lower limb dysmetria (uni- or bilateral), 4 = dysarthria

further neurological findings: 1 = saccadic smooth pursuit, 2 = pathological head impulse test (bilateral, if unilateral, left or right side indicated), 3 = rigidity, 4 = postural tremor, 5 = impaired ankle reflexes and/or reduced vibration sense, 6 = positive Romberg’s sign, 7 = deviation of the subjective visual vertical (SVV)

ABC: Activities-specific Balance Confidence Scale (ranging from 0 to 100%, > 80 high level of physical function; 50–80 moderate level of physical function; < 50 low level of physical function); FES-I: Falls Efficacy Scale-International (total score range from 16, i.e., no concern about falling, to 64, i.e., severe concern about falling); FGA: Functional Gait Assessment (ranging from 0 to 30, with 30 being the best possible score)

*f* female, *m* male

^a^FGA score below the age-referenced norm [10]

^b^FGA testing aborted due to permanent instability

^c^Siblings

### Procedures and variables

All patients underwent the Functional Gait Assessment (FGA), a 10-item clinical gait performance evaluation (overall score ranging from 0 to 30, with 30 being the best possible score). Quantitative assessment of walking ability of patients and healthy controls was performed on a 6.7 m long pressure-sensitive gait carpet (GAITRite^®^, CIR Systems, Franklin, NJ, USA) with a sampling frequency of 120 Hz. Each participant completed a comprehensive gait assessment protocol comprising seven different speed, sensory, and dual-task walking conditions: walking (1) at preferred speed, (2) slow speed, (3) maximal speed, (4) with head reclination, (5) with eyes closed, (6) during cognitive dual task (calculatory serial 7 task), and (7) during motor dual task (carrying a tray). For each condition, a total of four trials were recorded. Walking performance and stability for each recording were characterized by the following spatiotemporal gait parameters: mean walking velocity (cm/s), stride length (cm), stride time (cm), percentage of double support phase (%), percentage of swing phase (%), base of support (cm) as well as the stride-to-stride variability of stride length, stride time, and base of support. Stride-to-stride variability was determined using the coefficient of variation (CV).

### Statistical analysis

Descriptive statistics are reported as mean ± SD. Differences in walking performance of patients and controls were analyzed using a repeated-measures analysis of variance and Bonferroni post hoc analysis with group (patients vs. healthy controls) and walking condition as factors. Possible associations between clinical findings, fall status, and the average walking performance of patients across conditions (gait parameters as described above) were examined by a point-biserial Pearson’s correlation. Results were considered significant at *p* < 0.05.

### Availability of data and material

Anonymized datasets are available upon reasonable request to qualified researchers.

## Results

### Patient characteristics

Clinically, all patients showed a saccadic smooth pursuit and clinical signs for a mild cerebellar ocular motor impairment were found in two-thirds of patients. Eighty-nine percent of OT patients showed postural instabilities indicated by a positive Romberg’s test as well as a reduced vibration sense or impaired reflexes of the legs. 61% of OT patients presented with a lower limb dysmetria and 44% showed a uni- or bilateral pathological head impulse test. All patients reported considerable impairments in physical function and daily activity (mean ABC score: 45.7 ± 23.4) and a moderate to severe concern about the risk to fall (mean FES-I score: 36.7 ± 10.9). Fall events within the last 6 months were reported by one-third of patients.

### Gait performance and quantitative gait analysis

**Fig. 1:**
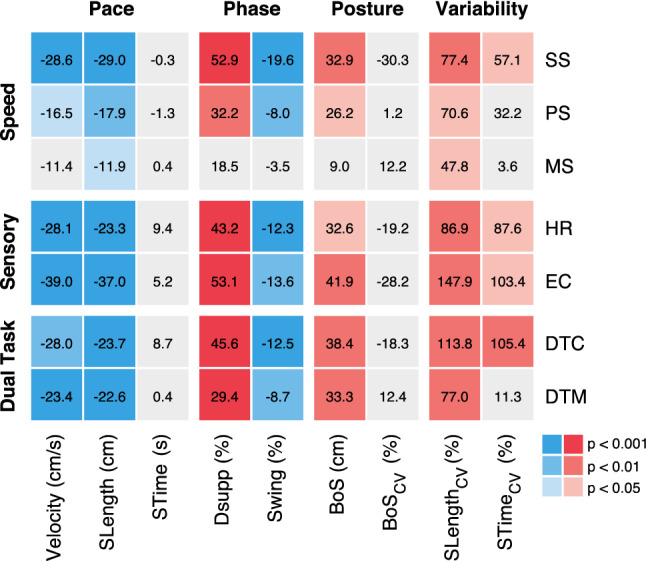
Overview of walking impairments in patients with orthostatic tremor. Comparison of walking performance in patients with orthostatic tremor vs. healthy controls across different speed, sensory, and dual-task conditions (rows) and with respect to different gait parameters (columns, grouped by four functional gait domains: pace, phase, posture, and variability). Numbers within each tile represent the mean percentage difference of patients’ walking performance compared to healthy controls. *SLength* stride length, *STime* stride time, *Dsupp* percentage of double support phase, *Swing* percentage of swing phase, *BoS* base of support, *CV* coefficient of variation, *SS* slow walking speed, *PS* preferred walking speed, *MS* maximal walking speed, *HR* head reclination, *EC* eyes closed, *DTC* cognitive dual task, *DTM* motor dual task

Functional impairments of gait during clinical assessment (FGA) were found in 50% of patients. Three patients were not able to perform the FGA due to permanent instability and six patients yielded a score below the age-referenced norm [10]. An overview on gait impairments of patients during quantitative gait assessment is presented in Fig. 1.

**Fig. 2:**
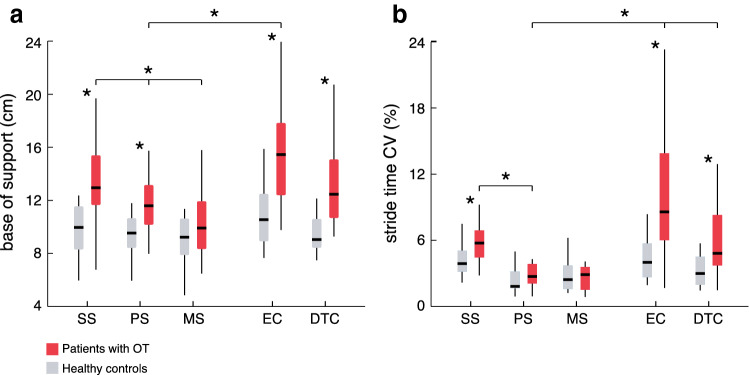
Modulation of gait characteristics across different walking conditions. Alterations in **a** base of support and **b** stride time CV in patients with OT (red) and healthy controls (grey) in dependence on the walking speed (SS, PS, MS) as well as during walking with EC or DTC. *Significant difference. *CV* coefficient of variation, *SS* slow walking speed, *PS* preferred walking speed, *MS* maximal walking speed, *EC* eyes closed, *DTC* cognitive dual task

During preferred walking, patients exhibited a broadened base of support (*p* = 0.018) with increased spatiotemporal gait variability (*p* = 0.010). Walking speed was moderately reduced (*p* = 0.026) with shortened stride lengths (*p* = 0.001) and increased periods of double support (*p* = 0.001). Gait impairments became considerably pronounced during slow walking but mainly disappeared during fast walking (*p* < 0.001). Withdrawal of visual feedback aggravated walking disability as did walking during cognitive dual task (*p* < 0.001, Fig. 2).

Walking impairments in patients were associated with clinical signs for a cerebellar ocular motor disorder (stride time *r* = − 0.56, *p* = 0.015; cadence *r* = 0.51, *p* = 0.033) and lower limb dysmetria (stride time CV *r* = 0.66, *p* < 0.01; velocity *r* = − 0.57, *p* = 0.013; stride length *r* = − 0.49, *p* = 0.04). Patients with a positive Romberg’s test showed increased gait fluctuations (stride time CV *r* = 0.47, *p* = 0.05). Furthermore, patients who had fallen within the recent past were found to walk at slower speeds (*r* = − 0.51, *p* = 0.030).

## Discussion

Considerable impairments of walking in patients with OT were apparent in clinical and quantitative assessment of gait. Instrumented gait analysis in these patients revealed a general slowdown of walking with specific pathological alterations, such as an increased base of support and increased spatiotemporal gait variability. Both aspects indicate a deficient regulation of dynamic postural stability and have been associated with an increased risk of falling [11–13]. These findings were reflected in the patients’ reports on limited physical activity and moderate to severe concerns about the risk to fall. Furthermore, one-third of patients did experience an actual fall within the past 6 months. Clinical examination in OT is, therefore, recommended to include a comprehensive examination of gait and physical function to identify those patients with a high risk for falling.

The staggering wide-based mode of walking in patients with OT is representative of the phenotype of a cerebellar or sensory ataxic gait disorder [14, 15]. Similar gait alterations have been reported in a recent publication [16]. Signs in our patients for cerebellar impairment coincide with previous reports [5, 17]. Additionally, recently it was hypothesized, that OT might primarily reflect a cerebellar pathology with altered functional connectivity between the cerebellum and supplemental motor areas as well as marked changes in cerebellar grey matter volume, which both correlated with clinical as well as tremor severity in patients [18–20]. In accordance to this hypothesis, application of a repetitive transcranial stimulation over the cerebellum led to a reduction of tremor severity and functional connectivity of these areas [18].

On the other hand, gait impairments in OT could also reflect deficits of proprioceptive sensing in the lower limbs, indicated by the frequent presentation of pallhypesthesia and a positive Romberg’s sign in our patients and the considerable aggravation of their gait instability during withdrawal of visual feedback. Accordingly, signaling by proprioceptive afferents has been suggested to become disrupted by the high-frequency tremor bursts within lower limb muscles [6] that persist in particular during the stance phases of walking [4].

Even though cerebellar and sensory ataxic gait disorders are both characterized by a broadened base of support and increased gait fluctuation during slow walking modes compared to self-selected walking [14, 21–23], a proprioceptive deficit as the primary source for gait impairments in OT is further supported by the observed normalization of walking with faster speeds at which gait regulation is known to become increasingly independent from sensory feedback cues [15, 24].

However, the gait disorder in patients with downbeat nystagmus syndrome—a disorder linked to dysfunction of specific vestibulo-cerebellar regions [25]—has been shown to closely resemble sensory ataxic walking impairments with a gait unsteadiness restricted to slow walking modes [26]. Thus, the prevalent finding of impaired cerebellar ocular motor function as well as related impairments in static postural control in our patients may also point to a specific vestibulo-cerebellar dysfunction underlying the gait disorder in OT (as signs of a midline cerebellar dysfunction).

Finally, we observed noticeable walking difficulties of patients with OT while performing a cognitive dual task. This supports recent evidence in cognitive processing deficits in OT that have been suggested to reflect an impaired functional frontocerebellar connectivity [27, 28]. The gait decline during dual-task walking is also observed in other neurological diseases with neurocognitive deficits, such as idiopathic normal pressure hydrocephalus [29–31].

In the end, the results of our study question the concept of OT as a primary tremor disorder and instead suggest a complex network disorder with a characteristic spino-cerebello-frontocortical gait disorder. Hence, the gait impairment in OT might directly reflect the pathological ponto-cerebello-thalamo-cortical dysfunction, which was previously described by a whole brain ^18^F-fluorodeoxyglucose-positron emission tomography analysis [5].

### Limitations

This study has several limitations. First, the OT patient cohort consisted of only 18 patients from one medical center. However, this sample size is similar to many previously published studies due to the rarity of disease with a low prevalence [5, 18, 32]. Second, while clinical signs for impaired ankle reflexes and/or pallhypesthesia were prevalent in our cohort, patients did not report further typical symptoms for a peripheral neuropathy, such as paresthesia, burning or tingling pain or progressive pareses. Within the context of the current study, we were not able to determine whether the reduced proprioceptive function is independent from or directly caused by OT. In favor of the latter option, it has been suggested that during prolonged standing in patients with OT, proprioceptive feedback from the periphery becomes increasingly synchronized at the tremor frequency [6]. This tremor-locking of proprioceptive afferents could disrupt normal peripheral feedback regulation of posture and give rise to an increased co-contraction of anti-gravity musculature leading to a vicious circle of escalating subjective and objective postural unsteadiness. This hypothesis has been further supported by the recently shown beneficial effects of a proprioceptive muscle tendon stimulation on tremor intensity and body sway in patients with OT [33]. However, further studies that particularly examine nerve conduction speed in OT patients in the presence and absence of tremor are necessary to conclusively decide on the origin of the prevalent proprioceptive dysfunction in these patients.

## Conclusion

Overall, patients with primary orthostatic tremor showed a gait disorder with broadened based of support and increased gait variability resembling a sensory or cerebellar ataxic gait. Gait performance declined during walking with eyes closed and improved during faster walking compared to healthy subjects indicating that proprioceptive deficits and/or a specific dysfunction within the vestibulo-cerebellum contribute to the gait impairment in OT. Furthermore, gait performance declined during cognitive dual task, which implies the presence of additional frontal processing deficits in patients with OT. These findings suggest that OT represents a complex network disorder associated to a specific spino-cerebello-frontocortical gait disorder that goes beyond mere tremor networks.
